# Opioids Use in Male Population Referred for Mandatory Urine Opioid Screen Before Marriage in Shiraz-Iran

**Published:** 2011

**Authors:** Jamshid Ahmadi, Mojtaba Naghshvarian, Ramin Afshari

**Affiliations:** 1 Research Center for Psychiatry and Behavioral Sciences**, **Shiraz University of Medical Sciences, Shiraz, Iran

**Keywords:** Iran, Opioid Use, Urine Opioid

## Abstract

**Objective:** To assess the prevalence of opioid abuse in a sample of male population in Shiraz, Iran.

**Methods:** A representative sample of 1219 subjects (aged 16 years or older) who were referred for urine opioid screen (UOS) as a mandatory test before marriage in Shiraz in 2005 were enrolled in this study.

**Results:** Mean age of the participants was 26.17 years (SD=6.12), ranging from 16 to 75 years. From them, 121 participants (9.93%) had positive urine opioid test. They were retested at least few days later by thin layer chromatography (TLC). The results showed that 22 (1.80%) had positive urine opioid test (based on TLC), whose mean age was 25.7 years, ranging from 19 to 32 years.

**Conclusion:** It can be concluded that at least 1.80% of men referred for UOS as a mandatory test before marriage used opioids. These results should be considered when preventive and therapeutic programs are being planned.

## Introduction

Opioid dependence is a psychiatric disorder (DSM-IV, American Psychiatric Association, 1994, and ICD-10, Classification of Mental and Behavioral Disorders) (1). Psychiatric disorders affect a significant part of the population, and substance abuse disorders are among the most common mental disorders (2). The Epidemiological Catchment Area (ECA) study, which involved personal interviews with 20000 individuals from five states in the United States, showed that just fewer than 14% of the population had alcohol use disorders some times during their lives (3). The national co-morbidity survey (4), carried out between 1990 and 1992, examined the extent of co-morbidity between substance use and other psychiatric disorders using a revised version of the Composite International Diagnostic Interview, CIDI: WHO. The National co-morbidity survey indicated that 14% of men and 5% of women met criteria for an alcohol use disorder in the last one year and 24% during their lifetime (5). In the national co-morbidity survey, men were more dependent than women and the lifetime prevalence of substance use disorders was 15% for men and 9% for women, and the one year prevalence of these disorders was 5% for men and 2% for women.

Although Iran had been an important opium producing center for many centuries, the situation has changed from 1955, when the first laws against the cultivation and use of opioids were introduced in this country. Iran was a signatory to the 1961 Convention on Psychotropic substance. So far, Iran has been an active member of the United Nation Commission on Narcotic drugs in the near and Middle East.

Despite the international links and control and treatment policies, drug addiction continued to be a serious problem. Large scale law enforcement activities resulted in seizure of considerable amount of drugs, there was a strict band on the cultivation of drug producing plants, and from 1974 responsibility for drug treatment was invested in National Iranian Society for the Rehabilitation of the disabled. This new organization, which benefited from substantial funding during a boom in the economy of the country, worked to improve inpatient facilities. However, such treatments became available to only a small proportion of the massive numbers of addicts. Subsequently, a major detoxification program was launched throughout Iran, placing more emphasis on outpatient treatment than on the hospitalization of addicts. Between 1974 and 1977, the National Iranian Society for the Rehabilitation of the Disabled had opened rehabilitation centers throughout the country to deal with 30,000 outpatients (6). However, limited publications are available due to evaluation of the success of this rehabilitation program.

Most of the research of drug abuse in pre-revolutionary Iran has been confined to studies of registered addicts in clinical settings, and there were no studies of young or other non-registered users. It is evident from these limited sources that, although opium had always been the most widely abused drug in Iran, the pattern had diversified in the period of the rapid growth of cities, population movement and the general economic changes, which characterized the decade prior to the revolution. Opioids hallucinogens and hypnotics were all reported as drugs of abuse among the clinical population studies. Besides, alcohol use increased substantially in the later pre-revolution years (6).

An official estimate of the size of the drug problem was made. In 1950 was claimed that approximately 7% of the total population was dependent on opium (6). The official drive against drug production and consumption in the following decades has reduced the scale of the traditional pattern of opium use. Long term opium dependents were registered but these authorized users became some of the major opium suppliers. The rapid economic and social changes of the 1970s following increase in oil prices led to the “Westernization” of the pattern of the drug abuse and diversity of substances involved.

The Islamic Revolution in 1979 was much more than the replacement of the Shah’s government by a religiously led government. Virtually every aspect of public life was affected. Many of policies of the previous administration were altered or even reversed because they were considered un-Islamic, and the drug policy was not immune from such changes. At this time, the National Iranian Society for the Rehabilitation of the Disabled ceased to be in charge of drug treatment in this country.

Subsequently, some attempts have been made to limit cultivation and distribution of drugs. The new regime made alcohol a prime target and provided a new national campaign against drug abuse. During the early months of 1980, the campaign became much stricter with extensive use of the death penalty for drug trafficking.

Two studies from this period indicated the nature of the problem at early stage of the revolution. Dalvand interviewed 200 newly registered addicts at the rehabilitation center in the major provincial capital of Shiraz (7), and Agahi who conducted a survey in a sample of the adolescents of Isfahan (8).

The first study showed that the clinics experienced a broader social range of addicts after the revolution and the aforementioned action of the authorities brought many of recently addicted individuals to the clinics. Heroin abuse predominated among those who were urban residents, whereas villagers attending the clinics were more likely to be opium users.

One aspect of the pattern, which seemed stable in Iran for many years, was the age of initiation. Nearly 80% of the sample, whether recent or long stabilized users, had started to use drugs regularly in their 20s or later, whereas in many western studies the equivalent percentage of late starters was much lower (9). In another survey of the adolescent population, 11% claimed to have ever used any drug. Opium users were more than hashish users (10).

After the above mentioned studies, there are few reported empirical studies conducted in Iran, and we have only official press announcement on the extent and success of the campaign drug to go on. The country’s economy and social order have been considerably affected by the war against Iraq and one can surmise that the drug program has been affected as much as most other aspects of life in the country. Therefore, Iran urgently requires baseline data about opium addiction such as motivation of drug use to provide guidance for policy making on prevention, treatment, and education about drug use.

At present, very little is known about substance dependence, especially opioid dependence, in Iran. Iranian drug policy states that the penalties for possession and use of all illegal substances are arrest and imprisonment. Alcohol is both religiously and legally prohibited. Illicit substances are opioid, alcohol, cannabis, stimulants, LSD, cannabis and hallucinogens.

The majority of the Iranian population is below than 25 years (about 60%) and a large proportion of these are students. In a study investigating the rate of substance use in Iranian senior high school students, 14% of boys reported using cigarettes and 5.7% were currently using other substances (11). In a research about substance abuse among Iranian university students, 24% of the students reported using a substance sometimes in their lives. The rate of substance use has been found to be higher in men than women (12).

This paper evaluated opioid abuse in a sample of male population in the Iranian city of Shiraz.

## Methods and Materials

*Subjects:* The population of this study included all 1219 males who referred to Valfajr clinic’s authorized laboratory in all working days for mandatory test from Shiraz city, the capital of Fars province, with a population of about 1.5 million in early 2005 (simple sampling).

*Procedure:* The data were collected from 1219 male subjects using urine drug screening tape test for opioid abuse (UOS) in governmental Center of Valfajr, and under close observation. Those with positive UOS were retested at least few days later by thin layer chromatography (TLC) to rule out false positive results and discriminate opium and heroin from medications misuse such as codeine and tramadol. We got permission from authorities of the clinic, and the urine results were anonymous.

*Analysis:* The data were analyzed using SPSS software version 12. Descriptive statistics were used to analyze the data. P-value < 0.05 was considered statistically significant.

## Results and Discussion

All of the participants were males whose mean age was 26.17 (SD=6.12), ranging from 16 to 75 years. From all subjects, 16 (1.31%) were illiterate, 189 (15.5%) had an education at the level of primary school, 398 (32.65%) secondary school, 407 (33.39%) high school, and 209 (17.15%) had higher education. Besides, 121 (9.93%) participants had positive urine opioid test. Educational level of all participants has been shown in table 1.

**Table 1:** Educational level of the participants

	Positive UOS †	Positive TLC ‡	Negative UOS †	Total
Illiterate	4 (0.33%)	1 (0.08%)	12 (0.98%)	16 (1.31%)
primary school	20 (1.64%)	5 (0.41%)	169 (13.86%)	189 (15.50%)
secondary school	42 (3.44%)	7 (0.57%)	356 (29.20%)	398 (32.65%)
high school	38 (3.12%)	9 (0.74%)	369 (30.27%)	407 (33.39%)
Higher education	17 (1.39%)	0 (0%)	192 (15.75%)	209 (17.15%)

† Urine Opioid Screen

‡ Thin Layer Chromatography

In general, 1.80% of the studied population had used opioids. TLC testing for marriage, and job and driving license applications have revealed similar results in Iran previously. These figures were 1.34%, 1.58% and 3.96% respectively (13). All of figures may be underestimated because the dating of urine collection was up to the clients and they may have refrained from opioid use temporarily before the test (14). Opioids are used in Iran (usually by opium pipe) for pleasure, as pain killer, and hypnotic and also for treatment of premature ejaculation. Moreover, many Iranian have false beliefs about opium and use this substance to cure their chronic diseases such as diabetes mellitus, hyperlipidemia and hypertension (15-17).

This research was confined to Shiraz, a large city located in the south of Iran. Hence, these findings should not be generalized to the whole Iranian male population, or even Shiraz city.

## Conclusions

It can be concluded that at least 1.80% of the male population studied in Shiraz showed positive opioid urine test results. This finding should be considered when preventive and therapeutic programs are being planned.

## Authors' Contributions

JA conceived and designed the evaluation and helped to draft the manuscript. MN participated in designing the evaluation and performed parts of the statistical analysis. RA revised the manuscript and performed the statistical analysis and revised the manuscript. All authors read and approved final manuscript.

## Notes

**Declaration of Interest:** None.
